# Statin Action Targets Lipid Rafts of Cell Membranes: GIXD/PM-IRRAS Investigation of Langmuir Monolayers

**DOI:** 10.1021/acs.jpcb.3c02574

**Published:** 2023-08-08

**Authors:** Michalina Zaborowska, Marcin Broniatowski, Philippe Fontaine, Renata Bilewicz, Dorota Matyszewska

**Affiliations:** † Faculty of Chemistry, University of Warsaw, Pasteura 1, 02093 Warsaw, Poland; ‡ Faculty of Chemistry, Jagiellonian University, Gronostajowa 2, 30387 Kraków, Poland; § Synchrotron SOLEIL, L’Orme des Merisiers, Départementale 128, 91190 Saint-Aubin, France; ∥ Faculty of Chemistry, Biological and Chemical Research Centre, University of Warsaw, Żwirki i Wigury 101, 02089 Warsaw, Poland

## Abstract

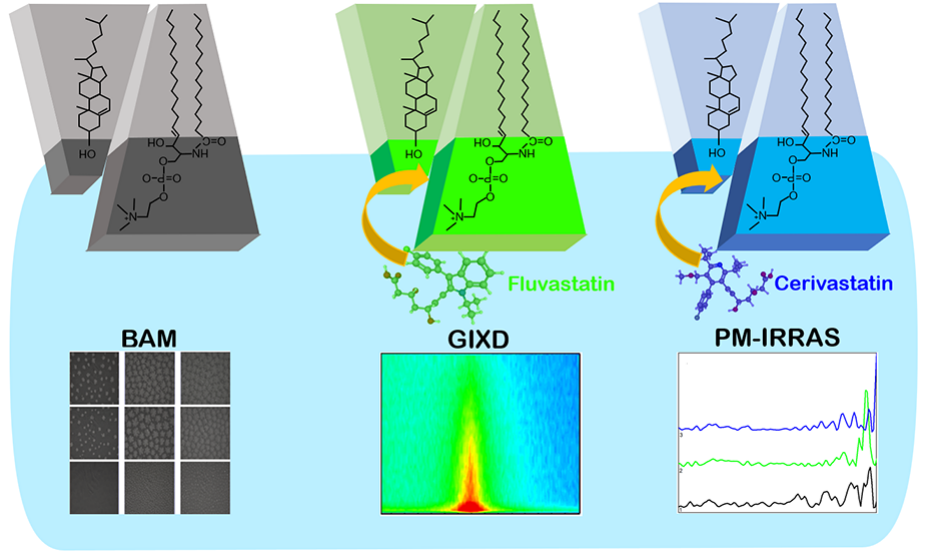

Lipid rafts are condensed regions of cell membranes rich in cholesterol and sphingomyelin, which constitute the target for anticholesterolemic drugs - statins. In this work, we use for the first time a combined grazing-incidence X-ray diffraction (GIXD)/polarization modulation infrared reflection absorption spectroscopy (PM-IRRAS)/Brewster angle microscopy (BAM) approach to show the statin effect on model lipid rafts and its components assembled in Langmuir monolayers at the air–water interface. Two representatives of these drugs, fluvastatin (FLU) and cerivastatin (CER), of different hydrophobicity were chosen, while cholesterol (Chol) and sphingomyelin (SM), and their 1:1 mixture were selected to form condensed monolayers of lipid rafts. The effect of statins on the single components of lipid rafts indicated that both the hydrophobicity of the drugs and the organization of the layer determined the drug–lipid interaction. For cholesterol monolayers, only the most hydrophobic CER was effectively changing the film structure, while for the less organized sphingomyelin, the biggest effect was observed for FLU. This drug affected both the polar headgroup region as shown by PM-IRRAS results and the 2D crystalline structure of the SM monolayer as evidenced by GIXD. Measurements performed for Chol/SM 1:1 models proved also that the statin effect depends on the presence of Chol–SM complexes. In this case, the less hydrophobic FLU was not able to penetrate the binary layer at all, while exposure to the hydrophobic CER resulted in the phase separation and formation of ordered assemblies. The changes in the membrane properties were visualized by BAM images and GIXD patterns and confirmed by thermodynamic parameters of hysteresis in the Langmuir monolayer compression–decompression experiments.

## Introduction

**Figure 1:**
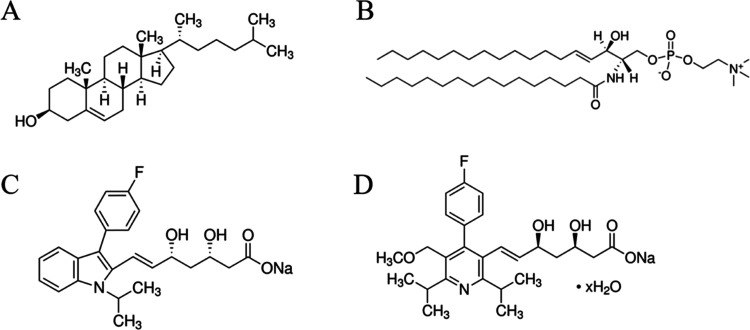
Structural formulas of lipids: (A) Chol; (B) SM, and statins: (C) FLU and (D) CER.

The formation of model biological membranes at the air–water interface by means of the Langmuir technique provides a convenient method to investigate the effects of drugs, toxins, and other substances ingested into the body. The biological membrane consists mainly of lipids, proteins, and sugar residues, which together form a fluid mosaic.^1,2^ However, the presence of ordered microdomains called lipid rafts was also shown.^3−5^ They are mostly composed of cholesterol (Chol) and sphingomyelin (SM) (Figure 1).^6,7^ Due to their surface properties, these lipids are responsible for the increased ordering in raft microdomains, which is partly caused by the formation of hydrogen bonds between the hydroxyl group of Chol and the amide group of sphingosine. It may also lead to the presence of surface complexes between these two types of molecules.^8,9^ The stability of such microdomains is additionally increased by the hydrophobic interactions between the sterol rings of Chol and SM acyl chains^10,11^ and results in the formation of a liquid-ordered phase (*L*_o_).^12^ Interactions in lipid rafts are also enhanced by the presence of phospholipids with unsaturated fatty acids (e.g., containing oleyl fatty acid residues, DOPC),^13,14^ which are responsible for the formation of disordered domains (*L*_d_) providing the fluid matrix for the ordered microdomains.^13,15−18^ On the other hand, some lipid models in the literature include saturated phospholipids (e.g., DPPC),^19,20^ explaining the tighter packing of lipids in rafts by unbent fatty acid chains. Irrespective of the exact composition of the model systems, the presence of the ordered domains in the biological membranes is crucial and increases their organization, influences their permeability,^21,22^ and affects the lipid–protein interactions.^23^

It has been shown that the majority of proteins present in eukaryotic cells are located in biological membranes including lipid rafts. The situation is similar in the case of the location of 3-hydroxy-3-methyloglutaryl-coenzyme A reductase (HMGR), which is a transmembrane protein occurring in the lipid rafts in the membranes of the endoplasmic reticulum (ER).^24−27^ HMGR is an enzyme supporting the process of Chol synthesis in hepatocytes.^28^ Regulation of this process is necessary for the prevention and treatment of cardiovascular diseases^29^ and can be performed by means of statins.^28^ This large group of drugs includes molecules differing in the degree of hydrophobicity (Figure 1), which, in turn, may determine their ability to bind to and penetrate the lipid membranes. FLU is a half-hydrophilic and half-hydrophobic drug with the octanol–water partition coefficient log *P* equal to 4.50.^30^ The part with the aromatic substituent is hydrophobic (more lipophilic), and the short acyl chain has several hydrophilic molecular groups. This synthetic drug in the form of sodium salt is characterized by p*K*_a_ equal to 4.15.^31^ Additionally, it was shown that FLU crosses membranes by passive diffusion, thanks to the low molecular weight and the amphiphilic characteristics.^32^ Hydrophobic interactions between the acyl chains and the aromatic groups of FLU have been also demonstrated.^33^ CER has similar hydrophobic/hydrophilic properties to FLU as indicated by the value of log *P* = 3.40–4.15;^34^ this drug is also used in the form of the sodium salt. However, it is characterized by two p*K*_a_ values: the lower one corresponding to the carboxylic acid form (p*K*_a_ = 4.38) and the higher value to the pyridine residue (p*K*_a_ = 5.29).^35^ CER was withdrawn from the pharmaceutical market in 2001 as a result of the reported deaths due to the side effect of rhabdomyolysis.^36^ This may be caused by the ability of this drug to deeply penetrate the structure of membranes, where it ends up in the CH_3_-terminal ends of the hydrophobic chains.^37^

In this work, we describe the effect of the two selected statins on the interactions with the monolayers of model lipid rafts formed at the air–water interface by means of the Langmuir technique. The two characteristic lipids, SM and Chol were selected to investigate the influence of statins on the lipid raft model. In our previous studies,^38,39^ we proved the specific character of the DOPC/Chol/SM 1:1:1 model with an equimolar lipid content. We showed that only with this specific molar ratio was a strong phase separation indicating the formation of Chol–SM domains as well as the presence of strong interactions between the individuals was observed. In order to provide a more detailed description of the condensed domains of lipid rafts and to determine other factors influencing the statin–lipid raft interactions, we deliberately did not include in the model used in this study any phospholipids such as DOPC providing the fluid matrix. We focus and carefully examine the influence of the selected statins on the individual components forming the ordered microdomains as well as on the mixed Chol/SM layers. For the first time, the combination of BAM with GIXD and PM-IRRAS was employed, which allowed us to follow the changes in the crystal structure and the domain formation of the model lipid rafts upon exposure to statins and draw conclusions on the effectivity of the penetration of the model lipid rafts by these medicines. Additionally, the role of the Chol–SM complex formation within the mixed monolayer on the effectivity of statin penetration was also evaluated.

## Methods

### Materials

The lipids used in the experiments include cholesterol (Chol), which was purchased from Merck and egg sphingomyelin (SM) from Avanti Polar Lipids. High-purity organic solvents (HPLC grade) obtained from Merck (Darmstadt, Germany) such as chloroform (for Chol) and chloroform/methanol 4:1 v/v mixtures (for SM) were used to prepare lipid solutions. In order to ensure physiological conditions during the measurements, the PBS buffer in Milli-*Q* water (resistivity 18.2 MΩ, Millipore) with the concentration of 0.01 M and pH 7.4 was used as the subphase. High-purity ≥ 98% statins, sodium salts of fluvastatin, FLU and cerivastatin, CER were also purchased from Merck. Therefore, throughout the text the abbreviations FLU and CER refer to the drugs in their sodium salt form present in the experimental conditions used in this study (PBS buffer pH = 7.4). Statins dissolved in PBS buffer were investigated at the concentration of 10^–5^ M, which is comparable to commonly used concentrations in drug–lipid studies.^40−42^

### Langmuir Technique

The Langmuir method was used to prepare mimetic model membranes and to measure their surface properties. The measuring setup consisted of a Langmuir trough (7.5 cm × 32.5 cm = 243 cm^2^), two hydrophilic barriers made of Delrin, and a Wilhelmy microbalance (KSV Nima, Sweden), on which a filter paper measuring surface pressure with an accuracy of ± 0.1 mN/m was hanged. The lipid solutions were spread on the precleaned subphase (PBS buffer or PBS buffer containing statins) by a Hamilton syringe. The measurement was started after 10 min of solvent evaporation with the barriers moving at the speed of 10 mm/min (corresponding to 75 mm^2^/min). Experiments were carried out at room temperature (21 ± 1 °C), and each measurement was repeated at least three times to ensure the reproducibility of the results. Therefore, the results shown in the figures represent the average of at least three measurements, and all calculated values of the parameters are reported together with their errors.

In order to compare the elastic properties of the monolayers, the compression modulus, reciprocal of compressibility *C*_s_, was calculated according to the formula given below
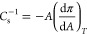
1where *C*_s_^–1^ is the compression modulus, *A* is the area per molecule, and π denotes the surface pressure. Based on the maximum value of *C*_s_^–1^, it is possible to characterize the phase of the monolayer. When the *C*_s_^–1^_max_ value is in the range of 0–12.5 mN/m, it is the gas phase (G). The range of 12.5–100 mN/m points to the liquid-expanded phase (LE), while the liquid-condensed phase (LC) is reflected by the maximum values within 100–250 mN/m and the solid phase (S) is above 250 mN/m.^43^

The Langmuir isotherms of single-component monolayers and mixed layers allow one to determine the forces acting between the components in the Chol/SM layer based on excess area parameters (*A*^Exc^).^44^ For this purpose, the following equations are used, where *A*_*i*_ denotes the area at the selected surface pressure of the monolayers of the individual components and *X*_*i*_ is their molar contribution to the mixed layer. The theoretical area *A*_12_^id^ of the Chol/SM 1:1 layer and *A*^Exc^ are described by the following equations:
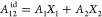
2
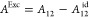
3*A*^Exc^ is calculated from the difference of the area per molecule for the mixed monolayer (*A*_12_) at the selected surface pressure and the theoretical area per molecule calculated from eq 2.

The multiple monolayer compression–expansion experiments were recorded for the binary Chol/SM 1:1 monolayers for two values of surface pressure: 30 mN/m corresponding to the biologically relevant surface pressure^45,46^ and 43 mN/m corresponding to the reorganization of the mixed monolayer. Based on the data obtained in repeated cycles, it was possible to calculate the thermodynamic parameters of hysteresis: the free energy of hysteresis (Δ*G*^hys^), the configurational entropy of hysteresis (Δ*S*^hys^), and the enthalpy of hysteresis (Δ*H*^hys^) according to the following equations^47−49^
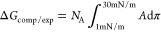
4
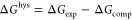
5
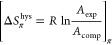
6
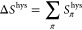
7
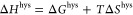
8where Δ*G*_comp/exp_ denotes the free energy of compression/expansion, *N*_A_ is the Avogadro number, and *R* is the gas constant. The values of the thermodynamic parameters refer to the first compression–expansion cycle.

### Grazing-Incidence X-ray Diffraction (GIXD)

The GIXD experiments were performed at the SIRIUS beamline in SOLEIL synchrotron (Gif-sur-Yvette, France) equipped with the liquid surface diffractometer and the Langmuir trough (R&K GmBh electronics, Germany) installed on the goniometer. The energy of the incoming X-ray beam was equal to 8 keV (λ = 1.55 Å). In order to protect the phospholipid monolayer from damage and reduce scattering, the Langmuir trough was sealed in a gastight box, which was flushed with helium. The GIXD experiments were performed for the phospholipid monolayers compressed to a target surface pressure of 30 mN/m, which was held constant throughout the experiment. The scattered signal was detected by a Pilatus3 1 M 2D pixel detector (Dectris Ltd., Switzerland) associated with a Soller collimator (JJ-X-ray Denmark) leading to a resolution of approximately 0.006 Å^–1^. The spectra were obtained by scanning the in-plane 2θ angle. At each point, the vertically scattered intensity was recorded to obtain the intensity map *I(Q*_XY_,*Q*_Z_*)*, where *Q*_XY_ and *Q*_Z_ are the components of the scattering vector defined by formulas 9 and 10, 2θ_XY_ is the angle between the incident and diffracted beams projected onto the horizontal plane, and α_f_ indicates the beam exit angle.^50−54^
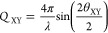
9
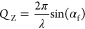
10The crystalline phases and unit cell parameters are evidenced by the peaks in the *I*(*Q*_XY_) dependence and the ratio of their intensities. Additionally, the *Q*_Z_ parameter defines the direction in which the intensity is collected, while the distribution of the intensity over *Q*_Z_ and *Q*_XY_ describes the structure of the monolayer. The positions of the maximum intensities of the Bragg peaks (max *I*(*Q*_XY_)) allow one to determine the cell parameters based on eq 11
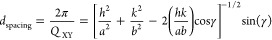
11where *d*_spacing_ denotes the repeat distance in the 2D lattice, *a* and *b* are lattice parameters, which can be correlated with the position of the Bragg peaks’ maximum, *h* and *k* are Miller indices, and γ is the angle between the lattice vectors. Based on the value of γ angle, three basic types of unit cells can be distinguished: hexagonal (γ = 120°), rectangular (γ = 90°), and oblique (90° < γ < 120°). Additionally, it is possible to determine the tilt angle denoted as τ, which indicates the deviation of the orientation of the molecules from a straight line perpendicular to the surface of the subphase^53^ (eq 12). In the following equation, ψ is the angle between the *Q*_XY_ vector and the tilt direction
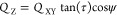
12Additionally, the area of the unit cell (*A*_uc_) may also be determined using eq 13
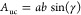
13An important parameter calculated from the dependence of the intensity on *Q*_XY_ is the full width at half-maximum (fwhm). Thanks to this, it is possible to determine the in-plane coherence length (*L*_XY_), which is related to the range of 2D crystallinity (eq 14).
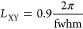
14The detailed construction of the diffractometer working at the SIRIUS beamline and the parameters of the synchrotron beam applied in the GIXD experiments are described on the SOLEIL Web site (www.synchrotron-soleil.fr), while further details of the GIXD technique can be found in the literature.^50^

### Polarization Modulation Infrared Reflection Absorption Spectroscopy (PM-IRRAS)

A Thermo Scientific PM-IRRAS (Nicolet iS50 FT-IR) spectrometer controlled by Omnic software and coupled to KSV software was used. The setup including an MCT-A liquid nitrogen cooled detector and a Langmuir trough (medium size trough, KSV Nima, Biolin Scientific, Sweden) was mounted on an optical table to provide the stability of the measurements and was protected by an enclosed Plexiglas cover assembly. The system was purged with dried air in order to ensure a constant vapor atmosphere. The spectra were collected in the range of 850–1800 cm^–1^ wavenumbers, corresponding to the region of the polar heads of phospholipids. The photoelastic modulator (PEM) was set to 1500 cm^–1^ in order to ensure its maximum efficiency in the polar headgroup region. The resolution of the measurements was 8 cm^–1^. The light beam (He-laser and IR) reached the surface of the monolayer compressed to 30 mN/m (corresponding to the physiological conditions) at an angle of 70°. The PM-IRRAS measurement is based on constant IR light modulation between the *p* and *s* polarization. *I*_s_ and *I*_p_ represent the reflectivity of *s* and *p* beams, respectively. The difference of the intensities (*I*_s_ – *I*_p_) provides surface-specific information, while the sum (*I*_s_ + *I*_p_) provides the reference spectrum. Therefore, the spectrum is defined as . Each measurement consisted of 512 scans, which were collected first for the pure subphase without a phospholipid monolayer (*S*_0_) and next for the phospholipid monolayer (*S*_π_). Before each measurement of the lipid monolayer spectrum, the background measurement of pure subphase was performed. Using the normalization procedure described by eq 15
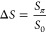
15the final spectrum (Δ*S*, after baseline correction) was obtained. *S*_π_ is the monolayer spectrum, and *S*_0_ is the background spectrum. Each measurement was repeated three times, and the results presented in the paper represent the average of these measurements.

### Brewster Angle Microscopy

The morphology of the layers was imaged by Brewster angle microscopy (BAM). The images were captured by using the Nanofilm Ep3 setup with an UltraBAM objective (Accurion, Germany). Each image represents an 800 μm × 430 μm field of view and was recorded with a lateral resolution of 2 μm. Images were taken during compression of the phospholipid monolayers at the air–water interface.

## Results and Discussion

### Interactions of Statins with SM Monolayers

**Figure 2:**
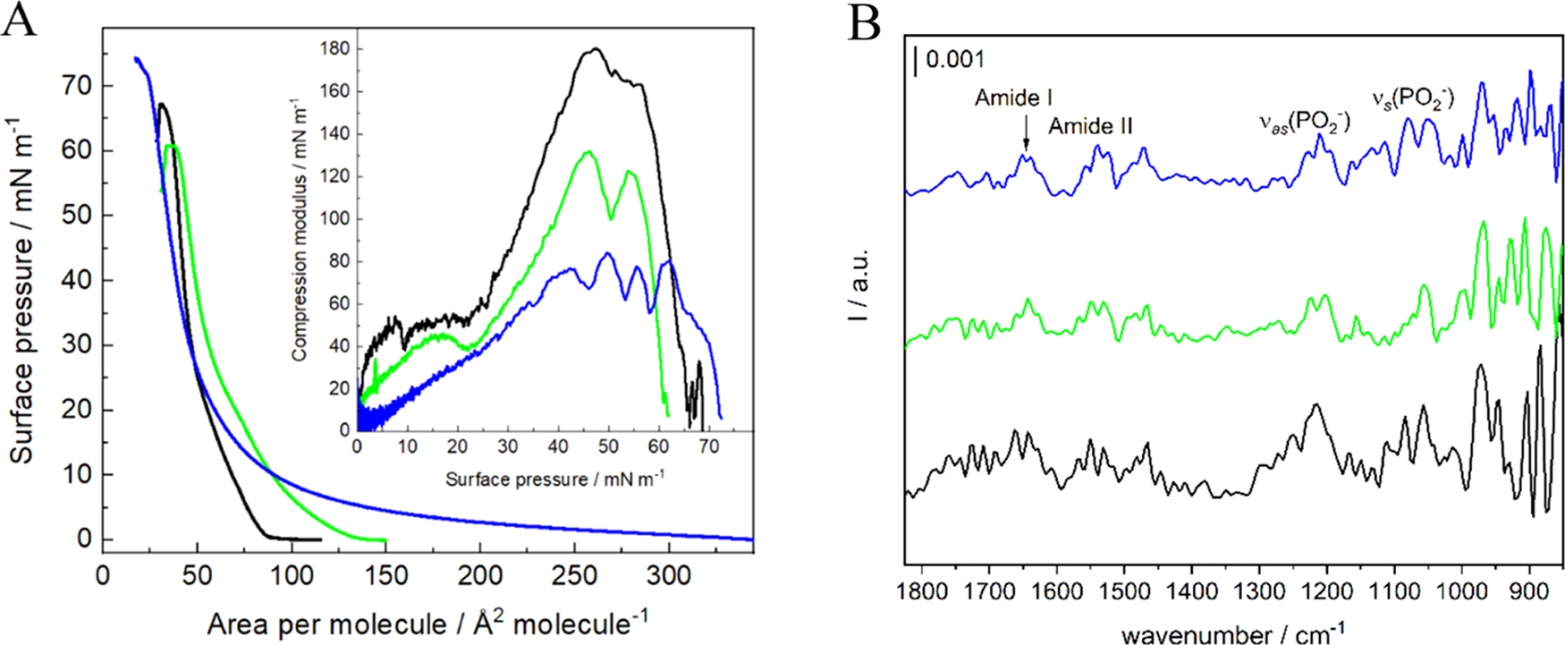
(A) Surface pressure–area per molecule (π–*A*) isotherms of SM monolayers; (B) PM-IRRAS spectra collected for SM monolayers compressed to 30 mN/m in the range of 1800–850 cm^–1^ on the pure PBS subphase (black) and PBS buffer containing 10^–5^ M FLU (green) and 10^–5^ M CER (blue). Inset in part (A): compression modulus vs surface pressure plot (*T* = 21 ± 1 °C).

Sphingomyelin is one of the main components of lipid rafts, and therefore, the influence of selected statins on its surface properties was investigated. SM monolayers prepared on PBS buffer pH = 7.4 form densely packed structures with the characteristic phase transition at around 20 mN/m clearly observed as a minimum on the *C*_s_^–1^–π plot (Figure 2).^29,55^ The area per molecule in a well-organized monolayer (*A*_0_) obtained by the extrapolation of the isotherm in its steepest part to the zero surface pressure is equal to 50.7 ± 1.0 Å^2^. The value of the compression modulus at the surface pressure of 30 mN/m corresponds to the liquid-expanded phase (∼85 mN/m), while the maximum value of the *C*_s_^–1^ falls into the liquid-condensed phase as it equals to 185 mN/m (Figure 2 and Table S1).^56^ This value decreases in the presence of statins in the subphase (Table S1). Although the most fluid layer is formed in the presence of CER, the higher *A*_0_ value is observed for FLU, which confirms the loosest packing of polar heads. This may indicate that FLU is located in or just behind the polar heads.^57,58^ On the other hand, the relatively high hydrophobicity of CER is supposed to allow this drug to effectively penetrate the SM monolayer from the beginning of the compression. However, at higher surface pressures, the isotherm of SM in the presence of CER crosses that of SM on pure buffer (Figure 2A). As a result, the area per molecule at 30 mN/m is almost the same for SM in both the presence and absence of CER in the subphase (Table S1), which might suggest the expulsion of the drug from the layer upon compression.

The PM-IRRAS studies provided information on the interactions within the polar headgroup region of SM monolayers exposed to statins. The IR spectra were collected in the 1800–850 cm^–1^ region corresponding to the SM polar headgroup vibrations (Figure 2B). Bands derived from symmetric and asymmetric vibrations of the O–C–C–N frame point to the *gauche* conformation of this part of the SM molecules,^59 ,60^ which is also influenced by the presence of both statins (Table 1). The shift of the asymmetric band toward lower wavenumbers suggests increased hydration of the choline group. It confirms the presence of interactions with the drugs dissolved in the subphase with this moiety of the SM molecules. Another important group is the phosphate group. According to the literature, asymmetric vibrations of this moiety are especially vulnerable to hydration and may result in the appearance of a band, which might be centered either at ∼1219 or ∼1250 cm^–1^ corresponding to hydrated and dehydrated PO_2_^–^ group, respectively.^60,61^ Following Hübner,^55^ the location of this band at 1214 cm^–1^ observed for SM monolayers formed on a pure buffer subphase (Table 1) confirms that the phosphate group is fully hydrated. The presence of FLU causes a slight shift of this band to a higher wavenumber. Additionally, the shoulder appearing at ∼1200 cm^–1^ might be attributed to the formation of intra- or intermolecular H-bonds between the phosphate group and the amine groups present in the choline moiety. This shoulder band also becomes well-developed in the presence of CER. Based on that, it can be concluded that for these two drugs, the increased dehydration of the P=O bond as well as the formation of hydrogen bonding takes place,^59,62^ which again confirms the interactions of statins with this part of the SM molecule.

**Table 1:** PM-IRRAS Band Position (in cm^–1^) for Sphingomyelin Monolayers Formed on Pure PBS Buffer and PBS Buffer Subphase Containing 10^–5^ M Concentrations of FLU and CER

band	SM	SM + FLU	SM + CER
amide I	1662	-	1650
	1643	1643	1639
amide II	1550	1550	1538
	1530	1530	1523
ν_as_(PO_2_^–^)	1214	1222	1211
		1203	
ν_s_(PO_2_^–^)	1083	-	1079
	1056	1056	1049
ν_as_(CN^+^(CH_3_)_3_)	971	968	968
ν_s_(CN^+^(CH_3_)_3_)	902	906	917
	883	875	898

The bands originating from the amide group distinguish SM from the other phospholipids. They are located at 1660 and 1640 cm^–1^ for the amide I band and at approximately 1550 and 1530 cm^–1^ for the amide II band.^63^ The position of the bands can point to a non- or weakly H-bonded amide group (higher frequency peak) or a stronger H-bonded amide group (lower frequency peak).^61,64^ Without the addition of the drugs in the subphase, both peaks are visible; therefore, there are both populations of SM molecules present (Table 1). The presence of FLU leads to the increased hydrogen bonding of the amide bonds,^65^ which is manifested by the presence of a single peak corresponding to the amide I band at a lower frequency. The interactions with CER lead to the overall shift of the amide peaks toward lower wavenumbers, which proves the increased formation of hydrogen bonding.

The last typical moiety contributing to the IR spectra in the polar headgroup region is C=O. However, in the structure of SM, contrary to other phospholipids, there is only one C=O group, which is part of the amide group. Therefore, the typical vibration band at approximately 1740 cm^–1^ is not present.^66^

**Figure 3:**
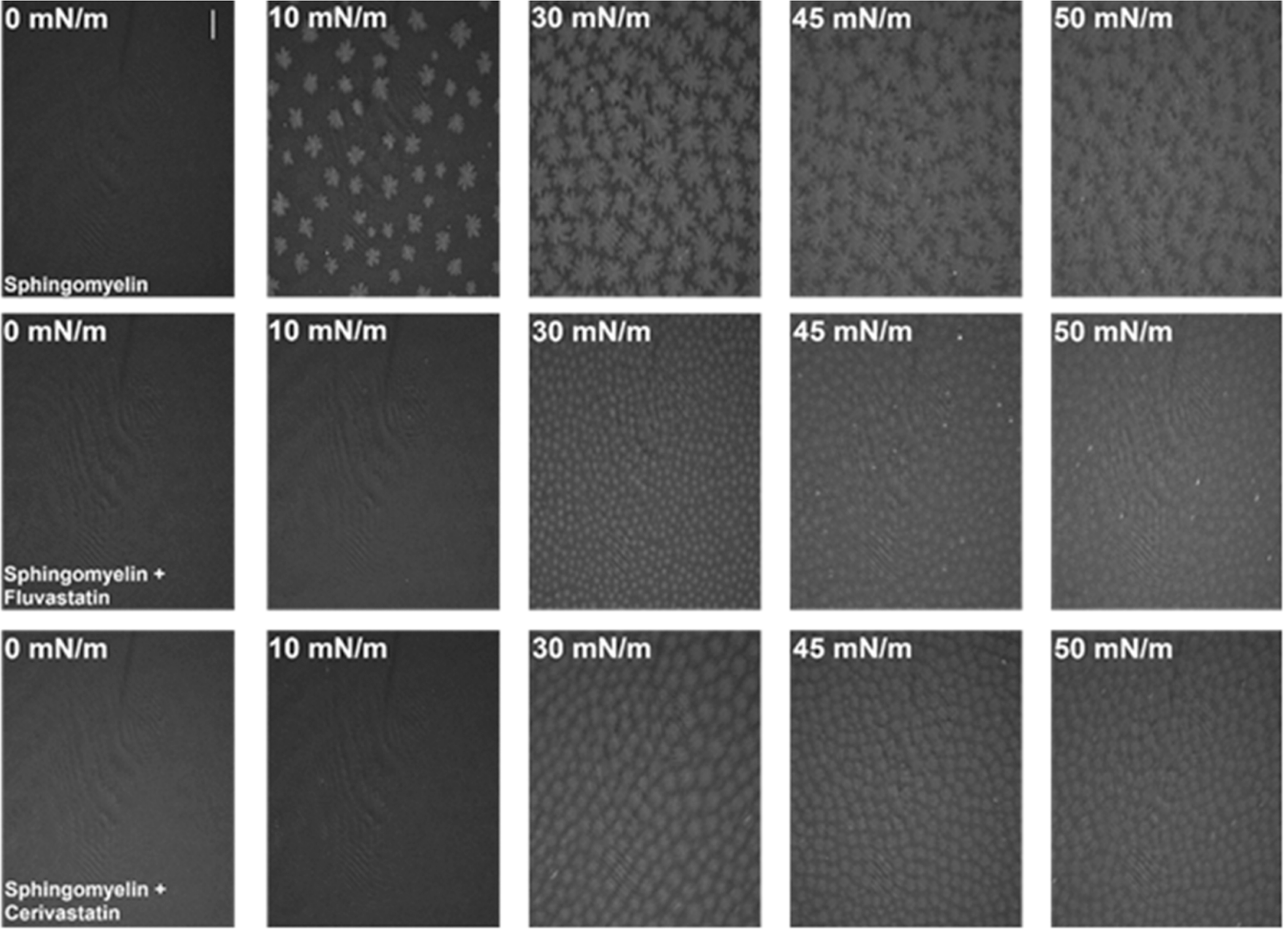
BAM pictures obtained at selected surface pressures for SM monolayers formed on PBS buffer pH 7.4 and PBS pH 7.4 containing 10^–5^ M statins. (*T* = 21 ± 1 °C). The scale bar is 100 μm.

BAM images captured for SM monolayers formed on pure buffer (Figure 3) show the formation of condensed SM domains, which start to occur at the surface pressure of approximately 10 mN/m.^67^ With the increasing compression of the layer, the domains merge and a more uniform, condensed monolayer can be observed. The presence of FLU and CER in the subphase shifts the formation of condensed domains to higher pressures (approximately 30 mN/m). The size of the domains is much smaller, and their shape is changed. It proves the stabilization of the liquid phase of the SM monolayers also shown by the decreased values of the compression modulus (Table S1). However, for both FLU and CER, the condensed domains, although altered, were still present. Therefore, if in the mesoscale observed by BAM the domains can be seen, it is worth checking the condensed nature of SM monolayers in the nanomolecular scale by means of GIXD.

**Figure 4:**
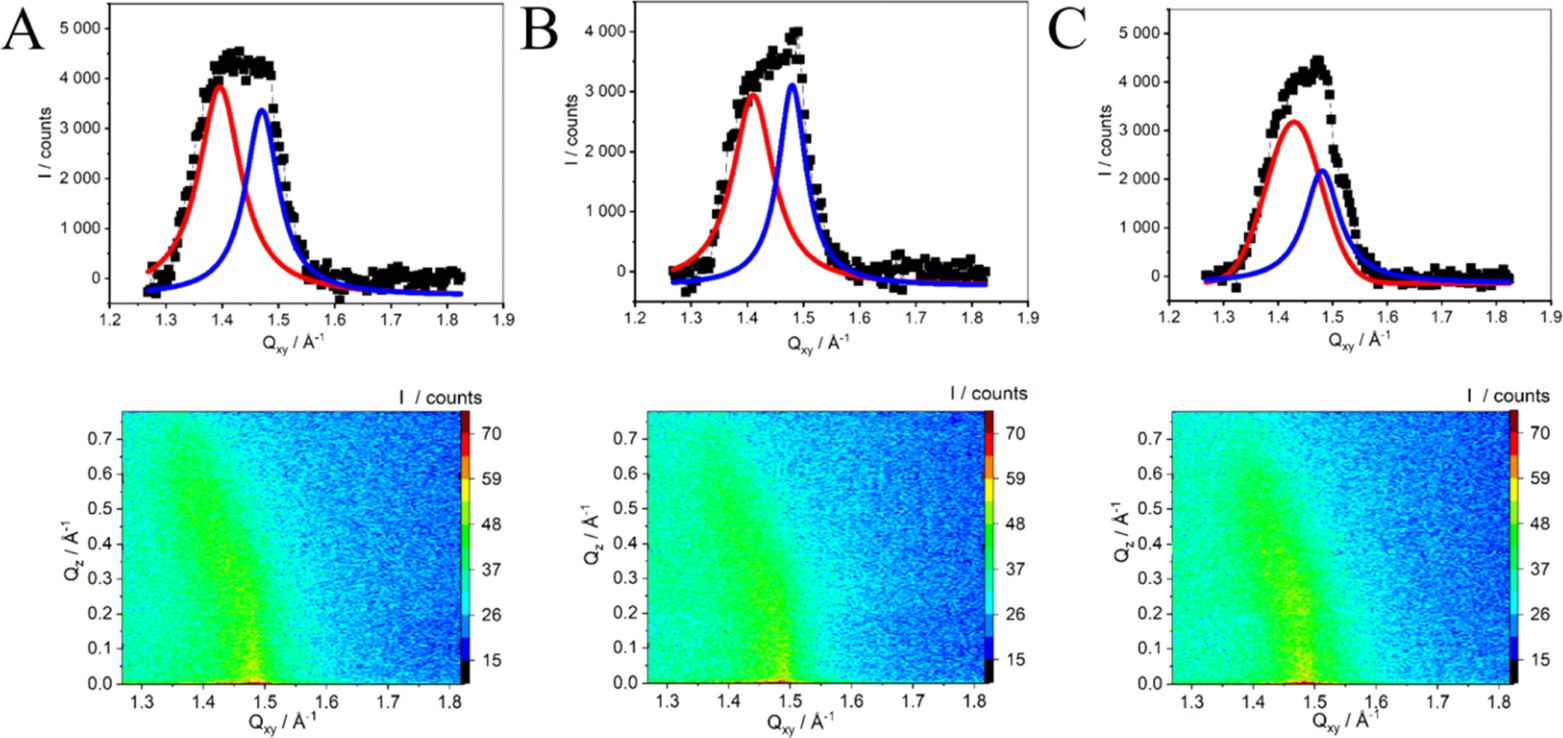
GIXD *Q*_z_-integrated Bragg peak profiles and corresponding *Q*_Z_–*Q*_XY_ intensity maps for SM Langmuir monolayers compressed to 30 mN/m on (A) PBS buffer, pH = 7.4 and PBS containing (B) 10^–5^ M FLU and (C) 10^–5^ M CER. Solid black points are experimental data, and red and blue lines are Lorentz curve fits.

GIXD profiles provided information about the organization of the acyl chains of SM monolayers in the absence and presence of selected statins (Figure 4) and therefore on the effectivity of the penetration of the SM monolayer by the drugs. The results obtained for pure SM monolayers were first compared with literature reports, which are somehow contradictory and therefore may cause confusion. The differences in the GIXD profiles are mostly due to the differences in the composition or temperature of the subphase. Ziblat et al.^12^ and Ratajczak et al.^68^ state that SM shows no Bragg peaks below 30 mN/m on a pure water subphase at 30 °C. It is attributed to the large hydrophilic headgroup of SM, which disturbs the molecular packing of hydrophobic moieties. Although the hydrophilic group does not participate in the crystalline packing,^12^ it plays a role in determining the structure of the SM monolayer. Other reports show one broad peak for SM monolayers compressed to 30 mN/m on pure water.^69^ It was attributed to the hexagonal lattice, and a possible moderate tilt of the molecules was also observed. On the other hand, in the other publication, stearoyl SM monolayers compressed to 30 mN/m on a pure water subphase exhibited an oblique lattice with acyl chains tilted from the monolayer normal with the intermediate tilt.^53^ Undoubtedly, the crystalline packing strongly depends on the composition of the subphase and the surface pressure at which the GIXD measurements are performed. In this case, SM monolayers were compressed to 30 mN/m on PBS buffer (*T* = 21 °C) and show two peaks, which suggests the presence of a rectangular unit cell. It can be compared with the information provided by Ratajczak et al.,^68^ who reported that SM monolayers formed on the pure water subphase at 30 °C revealed two peaks at 35 mN/m signifying the packing of tilted SM acyl chains in a distorted hexagonal unit cell. The reported *d* spacing was equal to *d*_(1,–1)_ = 4.29 and *d*_(0,1)+(1,0)_ = 4.61 Å.

Next, the effect of statins on the crystal structure of the SM monolayers was investigated. The presence of FLU and CER in the subphase leads to some changes in the Bragg profile and GIXD parameters compared to SM monolayers formed on pure buffer subphase (Table 2). The diffraction pattern remains the same, pointing to the rectangular cell. However, when FLU is added to the subphase, the position of Bragg peaks is slightly shifted toward bigger values. It is also accompanied by the slight changes in the parameters of the unit cell (*a* and *b*) and a decrease in the area of the unit cell (*A*_uc_) compared to SM monolayers on pure buffer together with the decrease in the tilt angle (τ). Similar mechanism was also observed for the interactions of polychlorinated biphenyls with DMPG crystalline domains.^70^ These results may also indicate that FLU might be to some extent included into the SM crystalline domains and may induce reorganization of SM molecules, which is also exhibited by the increasing correlation length especially in the <0,2> direction. The changes in the crystalline structure at the macroscopic level are also observed in BAM images (Figure 3). In the presence of FLU, the SM-condensed domains at 30 mN/m are significantly smaller and less developed compared to the SM monolayer formed on the pure buffer subphase. However, these domains, despite their reduced sizes, merge and form a uniform monolayer, which may be associated with the observed increased value of the correlation length.

**Table 2:** Characteristic GIXD Parameters of SM Langmuir Monolayers at 30 mN/m Formed on the Pure PBS Subphase and the PBS Subphase Containing 10^–5^ M FLU and CER

subphase	*Q*_xy_ (Å^–1^)	*Q*_z_ (Å^–1^)	*L*_xy_ (Å)	*d* (Å)	*a*,*b* (Å)	γ (^o^)	τ (^o^)	*A*_uc_ (Å^2^)
PBS	<0,2> 1.471	0	77 ± 5	4.27	5.295; 8.543	90	24.9	45.24
	<−1,1> 1.396	0.55	60 ± 3	4.50				
FLU	<0,2> 1.480	0	92 ± 6	4.25	5.235; 8.491	90	23.0	44.45
	<−1,1 > 1.410	0.51	63 ± 4	4.46				
CER	<0,2> 1.481	0	69 ± 9	4.24	5.141; 8.485	90	20.1	43.62
	<−1,1> 1.429	0.45	49 ± 3	4.40				

In the presence of CER, both Bragg peaks are shifted toward larger values; therefore, the parameters of the unit cell (*a* and *b*) and the *d* spacing are decreased. The area of the unit cell (*A*_uc_) and the tilt angle are smaller compared to those for SM monolayers on pure buffer. These changes are more pronounced than the ones observed in the presence of FLU. However, the values of the coherence lengths *L*_xy_ are smaller than those of pure SM monolayers, especially in the <−1,1> direction. It suggests that similarly to FLU, CER also penetrates the SM crystalline domains but the reorganization induced is of slightly different nature. This is also consistent with the BAM images obtained in the presence of CER, which show that the formation of condensed domains of SM is inhibited, but the domains are better developed and more separated from each other compared to the ones observed in the presence of FLU (Figure 3). On the other hand, the results of PM-IRRAS studies show a similar effect of CER on the polar heads of SM compared to FLU, while the Langmuir monolayer studies revealed that at 30 mN/m, the value of the area per molecule in the presence of CER in the subphase is similar to the one of SM monolayers formed on pure buffer (Table S1). Therefore, it may be concluded that CER interacts with SM monolayers by changing their organization, but upon increasing compression of the SM monolayer, it is partially expelled from the layer.

### Interactions of Statins with Chol Monolayers

**Figure 5:**
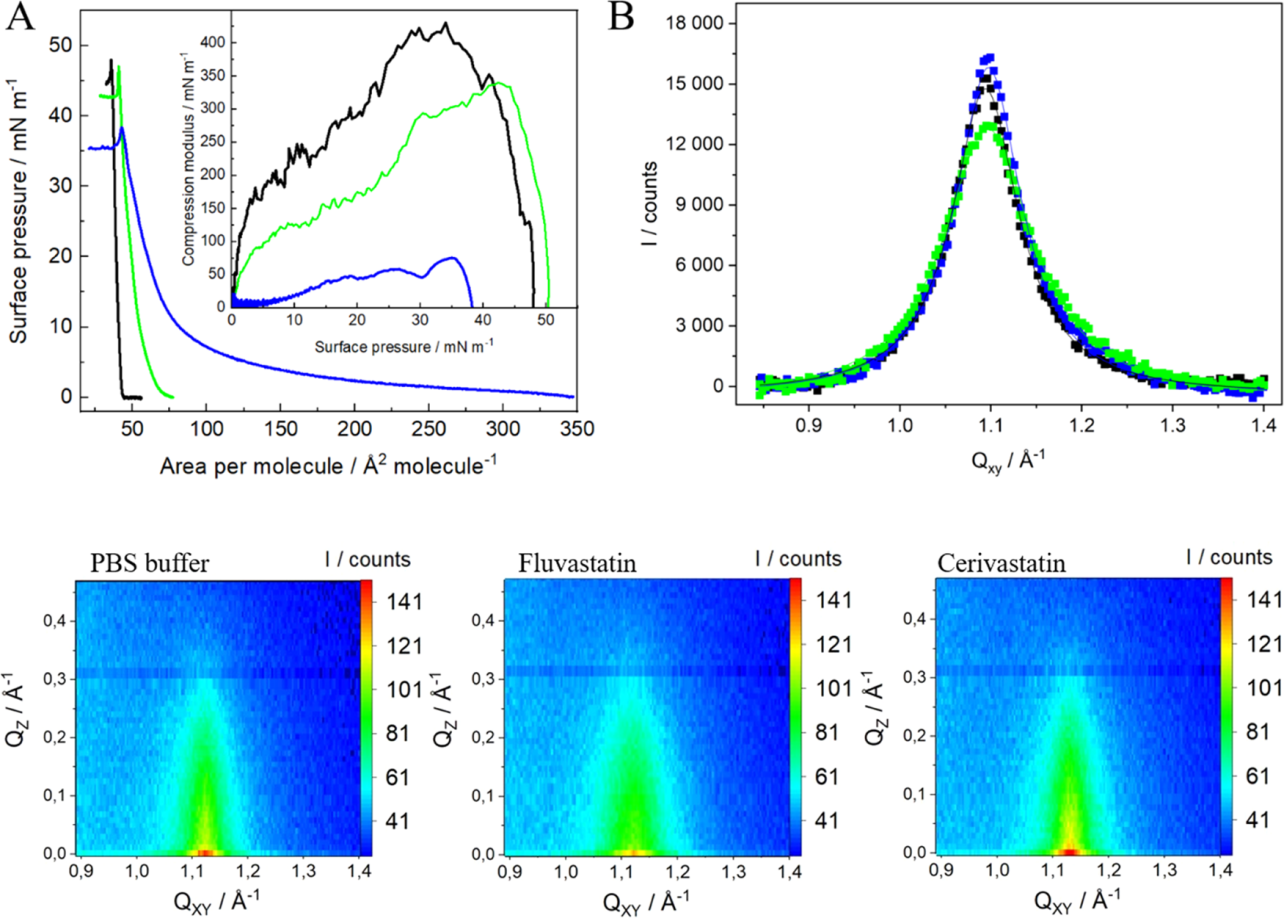
(A) Surface pressure–area per molecule (π–*A*) isotherms of Chol monolayers formed on PBS (black) and PBS containing 10^–5^ M of FLU (green) and CER (blue). Insets: compression modulus vs surface pressure plot (*T* = 21 ± 1 °C); (B) *Q*_Z_-integrated GIXD profiles for cholesterol Langmuir monolayers compressed to 30 mN/m on PBS buffer pH = 7.4 (black) and PBS containing 10^–5^ M FLU (green) and CER (blue); (C) *Q*_XY_*-Q*_Z_ intensity maps.

Chol is the second important component forming lipid rafts. Therefore, the interactions of statins with monocomponent Chol monolayers were also investigated. Cholesterol forms a rigid structure of tightly packed molecules at the air–water interface (*A*_0_ = 41.6 ± 0.4 Å^2^). The layers are characterized by a high value of the compression modulus (399 mN/m, Table S1) proving the solid phase.^9,71^ It collapses at the surface pressure of approximately 50 mN/m, which is preceded by the formation of the characteristic needle-like structures observed by BAM imaging at the surface pressure of 45 mN/m (Figure S1). In the presence of FLU, the organization of the layer does not change significantly, and the monolayer remains in the solid phase according to the maximum *C*_s_^–1^ value (Figure 5 and Table S1). However, a slightly lower homogeneity of the Chol layers in the presence of FLU is observed. Moreover, the needle-like structures observed at very high surface pressures are not as well-developed as in the case of pure Chol monolayers (Figure S1). The presence of CER in the layer leads to a significant increase in the fluidity of the monolayer, which is now in the LE (Table S1). Additionally, a substantial loosening of the structure of the layer is also observed (*A*_0_ = 72.7 ± 1.8 Å^2^). The presence of CER in the subphase also affects the stability of the Chol monolayer, resulting in the collapse, which occurs at a significantly lower surface pressure of approximately 37 mN/m (Figure 5A). Additionally, the BAM images captured just prior to the collapse (35 mN/m) do not show any typical needle structures but only the bright small spots (Figure S1). It may imply some aggregation of molecules just prior to collapse and thus its different mechanism. It also proves a significant fluidization of the Chol layer in the presence of CER. Such a large effect of the drug can be explained by the strong hydrophobic interactions between both components.

Additional information about the effect of statins on the hydrophobic part of Chol and thus on the packing and the 2D organization of monolayers was obtained by GIXD measurements performed for Chol monolayers compressed to 30 mN/m. Cholesterol forms monolayers with molecules arranged perpendicular to the air–water interface in the hexagonal lattice as proved by the only one diffraction signal observed on the diffraction spectrum at *Q*_xy_ = 1.09 Å^–1^ (Figure 5B). However, according to some literature reports, the packing of Chol molecules is better described by a trigonal lattice.^72,73^ These results together with the parameters of the crystal lattice (Table 3) stay in excellent agreement with the previous literature data.^42,73,74^ Interestingly, the presence of the two selected statins does not influence the 2D organization of the Chol monolayer in a significant way. Still only one diffraction peak is observed and the parameters concerning the unit cell remain unchanged (Table 3). However, the two drugs induce opposite changes in the correlation length (*L*_xy_). FLU leads to a smaller correlation length (46 Å), whereas CER leads to a comparable correlation length (62 Å) to Chol monolayers on pure buffer. Therefore, the effect of the drugs consists in the extend of the order or/and the amount of organized matter in the layer similarly as it was observed in the case of anthracycline drugs interacting with cholesterol monolayers.^42^ FLU interacts with the Chol monolayer in such a way that a smaller extent of order of the organized Chol domains is observed, which is consistent with the Langmuir monolayer studies and the values of the compression modulus (Table S1) as well as with the BAM imaging (Figure S1). Slightly different situation is observed for CER, which from the very beginning of the compression at low surface pressures easily penetrates the layer leading to higher lift-off and significantly lower values of the compression modulus. Surprisingly, such an effective participation of CER molecules into the air–water interface does not induce any significant changes in the extent of order of the organized Chol domains as shown by the lack of the changes in the correlation length values (*L*_xy_). Therefore, it may suggest that in the presence of CER, the Chol monolayer becomes inhomogeneous with liquid-expanded domains enriched in CER, which remain invisible for X-ray. Macroscopically, it results in a significant decrease in the compression modulus values (Table S1) and different BAM images obtained at higher surface pressures just before collapse compared to pure Chol monolayers (Figure S1). In the same time, in the presence of CER in the Chol monolayer, there are still ordered Chol domains, which can be detected by GIXD.

**Table 3:** Characteristic GIXD Parameters of Chol and Chol/SM 1:1 Langmuir Monolayers at 30 mN/m Formed on the Pure PBS Subphase and PBS Subphase Containing 10^–5^ M of Different Statins: FLU and CER

subphase	*Q*_xy_	*Q*_z_	*L*_xy_	*a = b;* γ	*A*_uc_
	(Å^–1^)	(Å^–1^)	(Å)	(Å); (^o^)	(Å^2^)
Chol					
PBS pH = 7.4	1.094	0	61 ± 1	6.63; 120	38.0
10^–5^ M FLU	1.097	0	46 ± 1	6.62; 120	37.9
10^–5^ M CER	1.097	0	62 ± 1	6.61; 120	37.8
Chol/SM 1:1					
PBS pH = 7.4	1.310	0	22 ± 1	5.54; 120	26.6
10^–5^ M FLU	1.312	0	24 ± 1	5.53; 120	26.5
10^–5^ M CER	1.318	0	25 ± 1	5.51; 120	26.2

### Interactions of Statins with Chol/SM 1:1 Monolayers

**Figure 6:**
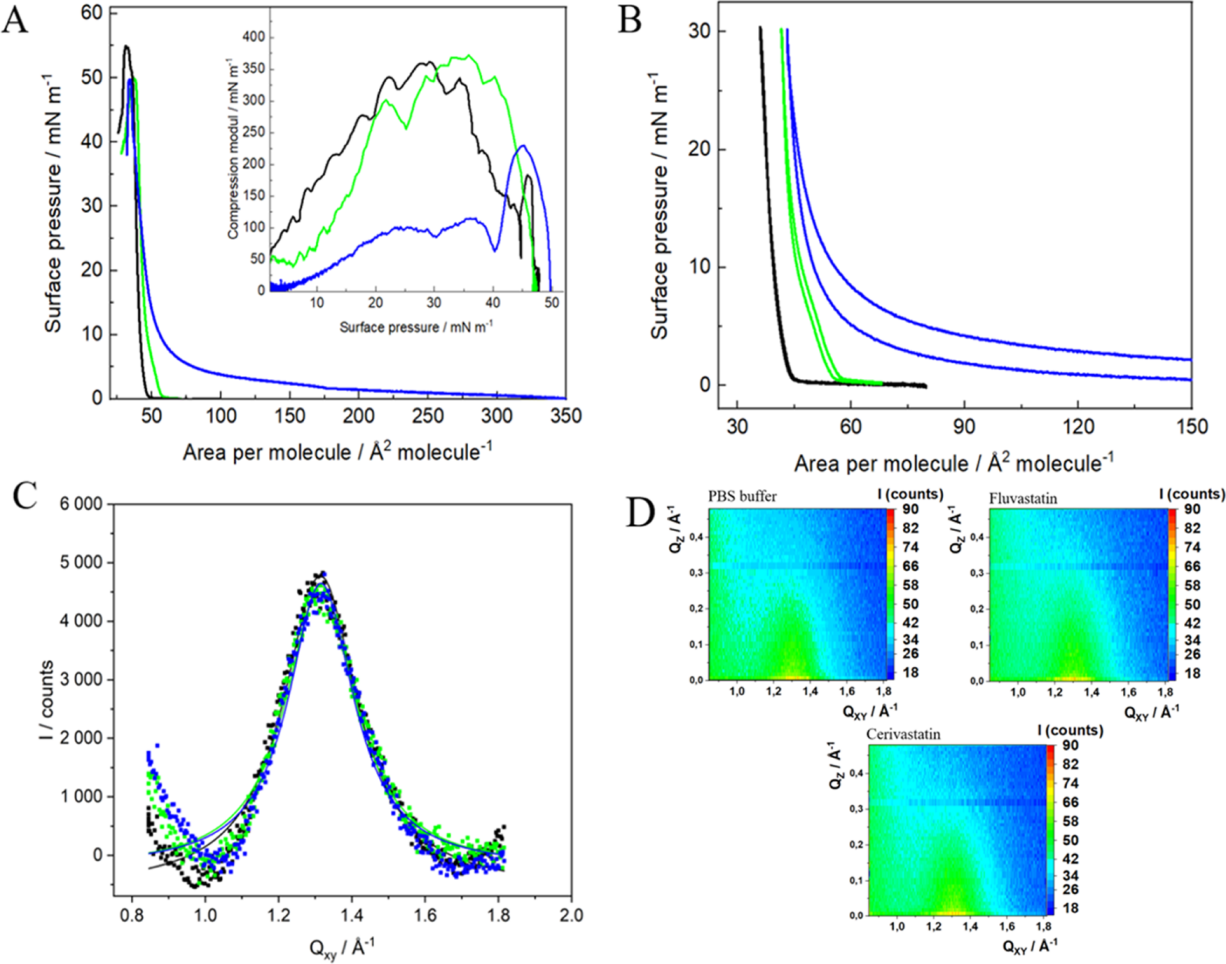
(A) Surface pressure–area per molecule (π–*A*) isotherms of Chol/SM 1:1 monolayers formed on PBS (black) and PBS containing 10^–5^ M of FLU (green) and CER (blue). Insets: compression modulus vs surface pressure plot (*T* = 21 ± 1 °C); (B) compression–expansion cycles for Chol/SM 1:1 monolayers on pure PBS buffer (black) and buffer containing 10^–5^ M FLU (green) and CER (blue) compressed to 30 mN/m; (C) *Q*_z_-integrated GIXD peak profiles for Chol/SM 1:1 Langmuir monolayers compressed to 30 mN/m on PBS buffer pH = 7.4 (black) and PBS containing 10^–5^ M FLU (green) and CER (blue). Solid lines represent the Lorentz fits; (D) *Q*_xy_–*Q*_z_ intensity maps.

It has been previously indicated that the main components capable of constituting ordered domains called lipid rafts include Chol and SM. Additionally, these two lipids are known to form stable complexes with the lipid ratio of Chol/SM 1:2.^68,69^ Therefore, in further studies, we have also investigated the effect of two statins on the two-component membranes. We have used the Chol/SM 1:1 model, which provides the presence of both the Chol–SM complex and the unbound Chol molecules but also reflects the SM-to-Chol ratio used in the previously employed three-component DOPC/Chol/SM 1:1:1 model system.^38,39^ In accordance with the characteristics of lipid rafts,^75^ the Chol/SM 1:1 mixture forms monolayers with tight lipid packing (*A*_0_ = 42.5 ± 0.5 Å^2^) and high level of organization typical for a solid phase (*C*_s_^–1^_max_ = 360 mN/m) (Figure 6A and Table S1). Based on the excess area parameters, one can conclude the predominance of attractive forces (*A*^Exc^ < 0, Figure S2) between the components in the mixed layer, which decreases with increasing surface pressure. This tendency may indicate privileged interactions between the components during the formation of the layer.

Interestingly, FLU does not change the elastic properties of the binary layer since the values of the compression modulus remain unchanged (Table S1). However, the isotherm in the presence of FLU is shifted toward larger areas per molecule, which implies that the drug is present in the monolayer causing its expansion. The more pronounced effect is observed for CER. The interactions with this drug lead to the expansion of the layer and the changes can be clearly observed especially at the beginning of compression. Based on the maximum value of the compression modulus, the phase of the 1:1 Chol/SM monolayer changes from solid to liquid condensed (Table S1). Nevertheless, at higher values of surface pressure (π > 35 mN/m), isotherms were recorded both in the presence and absence of CER converge, and thus one can conclude that the drug is removed from the layer. It is especially observed at the surface pressure of 40 mN/m, where it points to a reorganization causing further stiffening of the structure.

In order to further explore this observation, multiple compression–expansion cycles were recorded for the mixed Chol/SM 1:1 monolayers in the absence and presence of statins in the subphase. The monolayers were compressed to a surface pressure corresponding to the physiological conditions (30 mN/m). In all cases, three reproducible compression–expansion cycles were observed (Figure S2). However, only the first cycles, which were used for the calculation of the thermodynamic functions, are depicted (Figure 6). In the case of the measurements of hysteresis for Chol/SM 1:1 layers (without the addition of drugs), the values of thermodynamic functions such as the free energy of hysteresis (Δ*G*^hys^), the configurational entropy of hysteresis (*T*Δ*S*^hys^), and the enthalpy of hysteresis (Δ*H*^hys^) are close to zero (Table 4). It points to almost ideally elastic layers without the formation of any irreversible aggregates.^47−49^ It may mean that the formation of Chol–SM complexes within the layer is reversible upon the expansion of the layer. A similar situation is observed when FLU is present in the subphase, which means that this statin does not influence the reversibility of the formation of such complexes. It is also consistent with the π–*A* isotherms and the values of compression modulus showing a limited effect of FLU on the surface properties of mixed Chol/SM monolayers. However, in the presence of CER, the values of the thermodynamic functions become significantly negative. It may indicate that this statin, due to its increased hydrophobicity compared to FLU, is able to form more stable, irreversible arrangements within Chol/SM monolayers, which are not dispersed upon the expansion of the layer. The negative value of the free energy of hysteresis (Δ*G*^hys^) confirms the presence of cohesive intra- and intermolecular forces within the monolayer, while the negative value of *T*Δ*S*^hys^ proves the presence of entropically unfavorable interactions, which lead to the formation of more compact and ordered molecular organizations. It is possible due to the enthalpically favorable changes in the monolayer upon its compression, as indicated by the negative values of (Δ*H*^hys^). This tendency is even more pronounced when the binary monolayers are compressed to a higher surface pressure of 43 mN/m (Figure S3), just after the reorganization of the structure observed on the π–*A* isotherm. The values of the thermodynamic functions of hysteresis become even more negative in the presence of CER (Table S2).

**Table 4:** Thermodynamic Functions of Hysteresis: The Free Energy of Compression (Δ*G*_comp_), Expansion (Δ*G*_exp_), and Hysteresis (Δ*G*^hys^), the Configurational Entropy of Hysteresis (*T*Δ*S*^hys^), and the Enthalpy of Hysteresis (Δ*H*^hys^) for Chol/SM 1:1 Monolayers Formed on the Pure PBS Subphase and Subphase Containing Statins Compressed to 30 mN/m

subphase	Δ*G*_comp_ (kcal/mol)	Δ*G*_exp_ (kcal/mol)	Δ*G*^hys^ (kcal/mol)	*T*Δ*S*^hys^ (kcal/mol)	Δ*H*^hys^ (kcal/mol)
30 mN/m					
PBS pH = 7.4	0.13 ± 0.0	0.013 ± 0.0	–0.007 ± 0.003	–0.025 ± 0.01	–0.032 ± 0. 1
10^–5^ M FLU	0.19 ± 0.0	0.15 ± 0.0	–0.03 ± 0.0	–0.26 ± 0.02	–0.3 ± 0.03
10^–5^ M CER	1.74 ± 0.2	0.72 ± 0.0	–1.0 ± 0.1	–3.0 ± 0.1	–4.05 ± 0.1

The conclusion on the formation of ordered, irreversible assemblies within the Chol/SM 1:1 layer in the presence of CER drawn from the multiple compression–expansion cycles may seem to contradict the results of the surface pressure measurements and especially the calculations of the compression modulus showing a significant decrease of this parameter, which points to the formation of a more liquid layer (Figure 6). However, BAM images recorded for the binary layers reveal the phase separation of the Chol/SM 1:1 monolayer in the initial stages of the compression (0–15 mN/m) when CER is present in the subphase (Figure S4). At first, the black spots and then a visible division between the more and less ordered phases is observed. With further compression of the layer, some crystallites start to appear, and in the final stage of the compression large, densely packed domains are clearly visible. Interestingly, FLU has no such significant effect on the morphology of the Chol/SM layer as CER, which is consistent with Langmuir monolayer studies.

We also employed GIXD to obtain more detailed information on the crystal lattice of the binary model systems. Chol/SM 1:1 monolayers form well-ordered crystal assemblies and show only one diffraction peak corresponding to the hexagonal lattice (Figure 6C) characterized by the parameters presented in Table 3. The position of the single diffraction peak is observed at 1.310 Å^–1^, which is consistent with the results presented by Flasiński et al. for Chol/SM 1:1 monolayers^68,69^ and Ratajczak et al. for mixed Chol/SM monolayers of different ratios.^68^ It is evident that the obtained diffraction pattern is influenced by the presence of both components of the mixed layer. The rectangular arrangement of chains with next neighbor tilt azimuth observed for pure SM is changed to a hexagonal arrangement with vertical chains, which is more characteristic for Chol.^68^ However, the structure of pure Chol is not observed since the signal at 1.094 Å^–1^ typical for pure cholesterol monolayers is not present.^76^ Such a diffraction pattern proves the mixing of both components. In the presence of two selected statins, the position of the diffraction peak did not change significantly. On the other hand, the coherence length *L*_xy_ increases only slightly (Table 3), while the area of the unit cell decreases, especially in the presence of CER. It suggests that both FLU and CER lead to some changes in the range of the crystallinity within the two-component Chol/SM monolayer forming the complexes. This effect is more visible for CER compared to FLU, although in both cases, the observed changes are not that significant (Table 3). However, when considered together with the Langmuir monolayer, hysteresis, and BAM results, it shows the separation of the phases of the Chol/SM monolayers in the presence of CER, which leads to the formation of more ordered domains within such a monolayer.

## Conclusions

In this work, we focused on the effect of the selected anticholesterolemic drugs, fluvastatin and cerivastatin on the model lipid raft systems composed of sphingomyelin, cholesterol, and their equimolar mixture. The investigation of the influence of two selected statins on the single components of the lipid rafts revealed that apart from the hydrophobicity of the drug, the organization of the layer also determines the drug–lipid interactions. Sphingomyelin, which forms less organized monolayers, is strongly affected by both statins. Apparently, these drugs interact with the polar headgroup region, as shown by PM-IRRAS studies. As a result of those interactions, the 2D crystalline structure of the SM monolayer is also changed since the parameters of the unit cell and the tilt angle of acyl chains were altered. These results were also supported by BAM experiments, which allowed us to follow the changes in the morphologies of the layers in the mesoscale. It proves the ability of FLU to penetrate the SM layers deeper into the hydrophobic part. Similarly to fluvastatin, cerivastatin also participates in the SM crystalline domains, but the induced reorganization is of slightly different nature. Despite the effective penetration of the SM layer, which leads to the changes in the organization of the molecules, the increasing compression of the monolayer results in a partial removal of CER from the SM layer. In the case of cholesterol, which forms very compact, well-organized monolayers, in the presence of fluvastatin, the order of the organized cholesterol domains was affected to a lower extent, as evidenced by the values of the compression modulus and GIXD results. Cerivastatin, despite its effective penetration of the layer at the beginning of the compression, does not induce any significant changes in the order of the organized cholesterol domains detected by GIXD. It may suggest an inhomogeneity of the Chol monolayer exposed to this statin, with liquid-expanded domains enriched in cerivastatin remaining invisible for X-ray. Such changes in the morphology of the layers on the macroscopic level were also confirmed by the BAM images.

The formation of Chol–SM complexes in the binary Chol/SM 1:1 layer reported in the literature^68,69^ changes the action of statins. The presence of FLU leads to the disorganization of the Chol/SM monolayer observed in Langmuir studies, although this effect is not large. This can be explained by assuming that the presence of Chol–SM complexes prevents effective interaction of fluvastatin with the mixed monolayer. The observed changes are much more pronounced for CER. This hydrophobic statin leads to a significant fluidization of the binary monolayer, manifested by a decrease in the compression modulus values. However, the formation of irreversibly behaved assemblies in the presence of CER was indicated by the values of thermodynamic parameters calculated from the data obtained in multiple monolayer compression–expansion cycles. These effects may be attributed to interactions both with hydrophobic parts of cholesterol and sphingomyelin and also with the polar regions of the latter. The increased hydrophobicity of CER allows for a more effective penetration of the binary layer, even despite the formation of Chol–SM complexes, which seemed to prevent stronger interactions with the less hydrophobic fluvastatin. It may be suggested that the above-presented effects of statins on the structure of lipid rafts may contribute to the reported unwanted side effects of the statin therapy, especially for more hydrophobic drugs such as cerivastatin.

## Supporting Information Available

Additional calculations of characteristic parameters of Langmuir monolayers, BAM images of Chol and Chol/SM 1:1 monolayers, values of the excess area (*A*^Exc^) for Chol/SM 1:1 monolayers, compression–expansion cycles, and thermodynamic functions of hysteresis for Chol/SM 1:1 monolayers (PDF)
