# Supplementary material for: Statin Action Targets Lipid Rafts of Cell Membranes: GIXD/PM-IRRAS Investigation of Langmuir Monolayers
Source: J Phys Chem B. 2023 Aug 8;127(32):7135–47. doi: 10.1021/acs.jpcb.3c02574 (PMC10440791; doi:10.1021/acs.jpcb.3c02574)
Supplement: Supplementary file 1 — jp3c02574_si_001.pdf [file jp3c02574_si_001.pdf]

Supporting Information

Statin Action Targets Lipid Rafts of Cell Membranes: GIXD / PM-IRRAS Investigation of Langmuir Monolayers.

Michalina Zaborowska[1], Marcin Broniatowski[2], Philippe Fontaine[3], Renata Bilewicz[1] and Dorota Matyszewska[4*]

[1]Faculty of Chemistry, University of Warsaw, Pasteura 1, 02093 Warsaw, Poland

[2]Faculty of Chemistry, Jagiellonian University, Gronostajowa 2, 30387 Kraków, Poland

[3]Synchrotron SOLEIL, L'Orme des Merisiers, Départementale 128, 91190, Saint-Aubin, France

[4]Faculty of Chemistry, Biological and Chemical Research Centre, University of Warsaw, Żwirki i Wigury 101, 02089 Warsaw, Poland

Table S1 Characteristic parameters of Langmuir monolayers formed on PBS buffer and PBS subphase containing 10^{-5} M of statins.

	**Subphase**	**A_0 (Å^2)**	**$A_{\pi=30\ mN/m}$ (Å^2)**	**$C_s^{-1}max$ (mN/m)**
Chol	PBS pH=7.4	41.6±0.4	37.5±0.2	399±10
	10^{-5} M FLU	43.8±0.7	39.4±0.3	310±8
	10^{-5} M CER	72.7±1.8	48.0±2.0	67±12
SM	PBS pH=7.4	50.7±1.0	44.3±1.0	185±6
	10^{-5} M FLU	64.8±0.7	55.6±1.1	131±0
	10^{-5} M CER	59.7±2.0	45.1±0.2	87±4
Chol:SM 1:1	PBS pH=7.4	42.5±0.5	38.0±0.3	360±11
	10^{-5} M FLU	44.6±0.5	41.3±0.3	360±3
	10^{-5} M CER	50.0±0.0	40.2±1.0	220±10

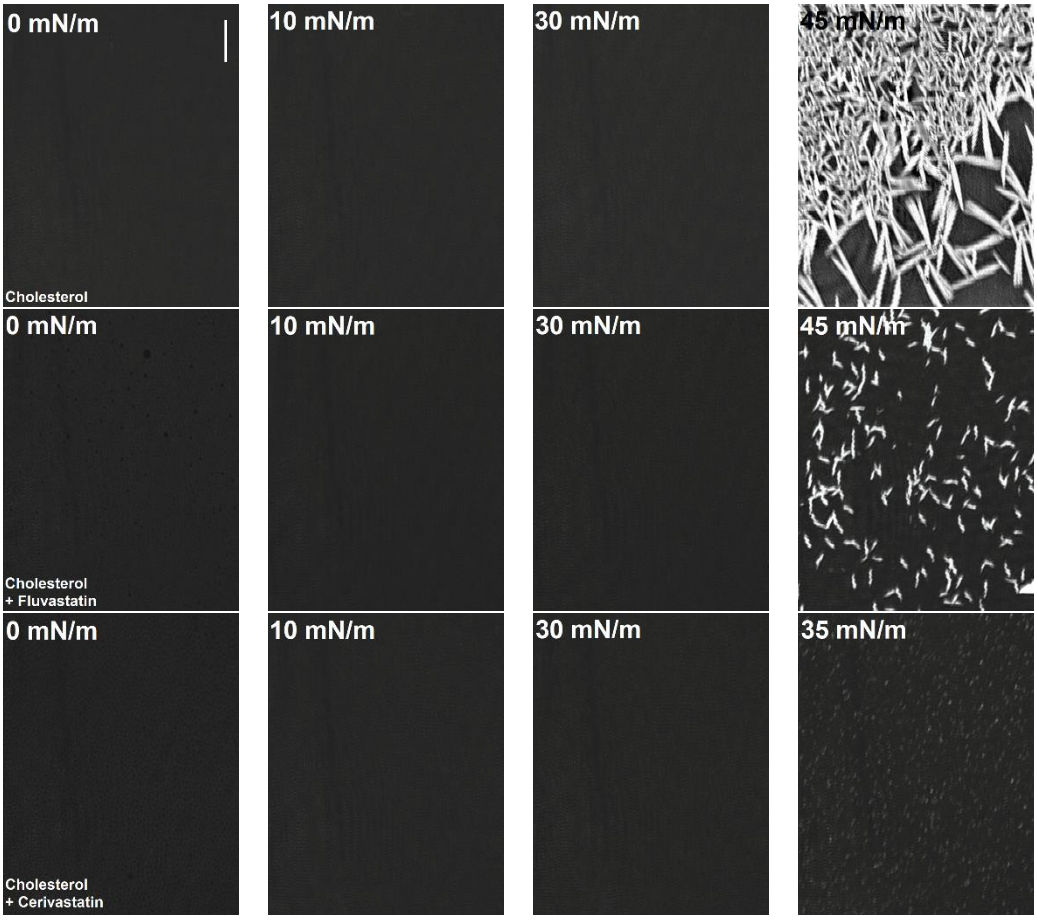

Figure S1. BAM pictures obtained at selected surface pressures for Chol monolayers formed on PBS buffer pH 7.4 and PBS pH 7.4 containing 10^{-5} M statins. (T=21±1°C). The scale bar is 100 μm.

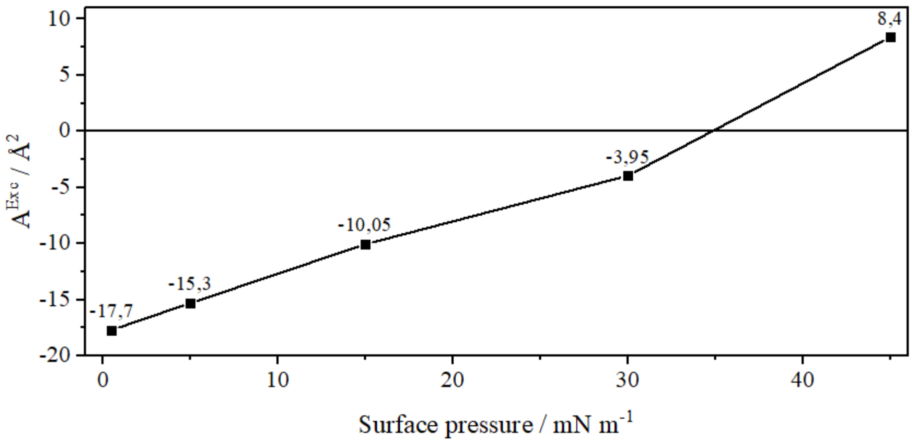

Figure S2. Values of the excess area (A^{Exc}) calculated at selected surface pressures for Chol:SM 1:1 monolayers.

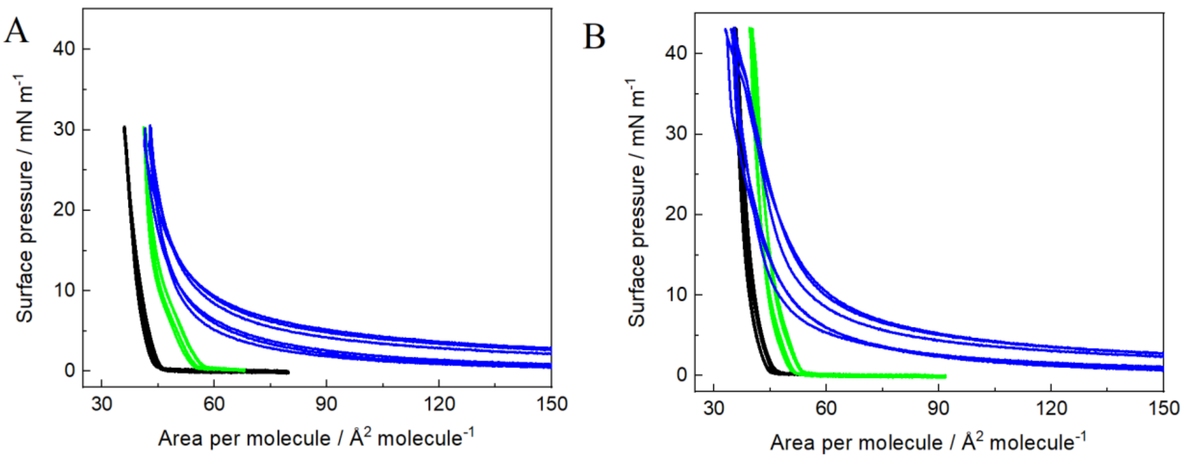

Figure S3 Compression-expansion cycles for Chol:SM 1:1 monolayers on pure PBS buffer (black) and buffer containing 10^{-5} M FLU (green) and CER (blue) compressed to A) 30 mN/m and B) 43 mN/m.

Table S2 Thermodynamic functions of hysteresis: the free energy of compression (ΔG_{comp}), expansion (ΔG_{exp}) and hysteresis (ΔG^{hys}), the configurational entropy of hysteresis ($T\Delta S^{hys}$), and the enthalpy of hysteresis (ΔH^{hys}) for Chol:SM 1:1 monolayers formed on pure PBS subphase and PBS subphase containing statins compressed to 43 mN/m.

Subphase	ΔG_{comp}/ kcal·mol^{-1}	ΔG_{exp}/ kcal·mol^{-1}	ΔG^{hys}/ kcal·mol^{-1}	$T\Delta S^{hys}$/ kcal·mol^{-1}	ΔH^{hys}/ kcal·mol^{-1}
			43 mN/m		
PBS pH=7.4	*0.17±0.0*	*0.13±0.0*	*-0.05±0.0*	*-2.4±0.2*	*-2.5±0.5*
10^{-5} M FLU	*0.26±0.07*	*0.17±0.0*	*-0.09±0.01*	*-1.5±0.2*	*-1.6±0.2*
10^{-5} M CER	*1.48±0.2*	*0.79±0.2*	*-0.7±0.02*	*-6.8±0.1*	*-7.5±0.3*

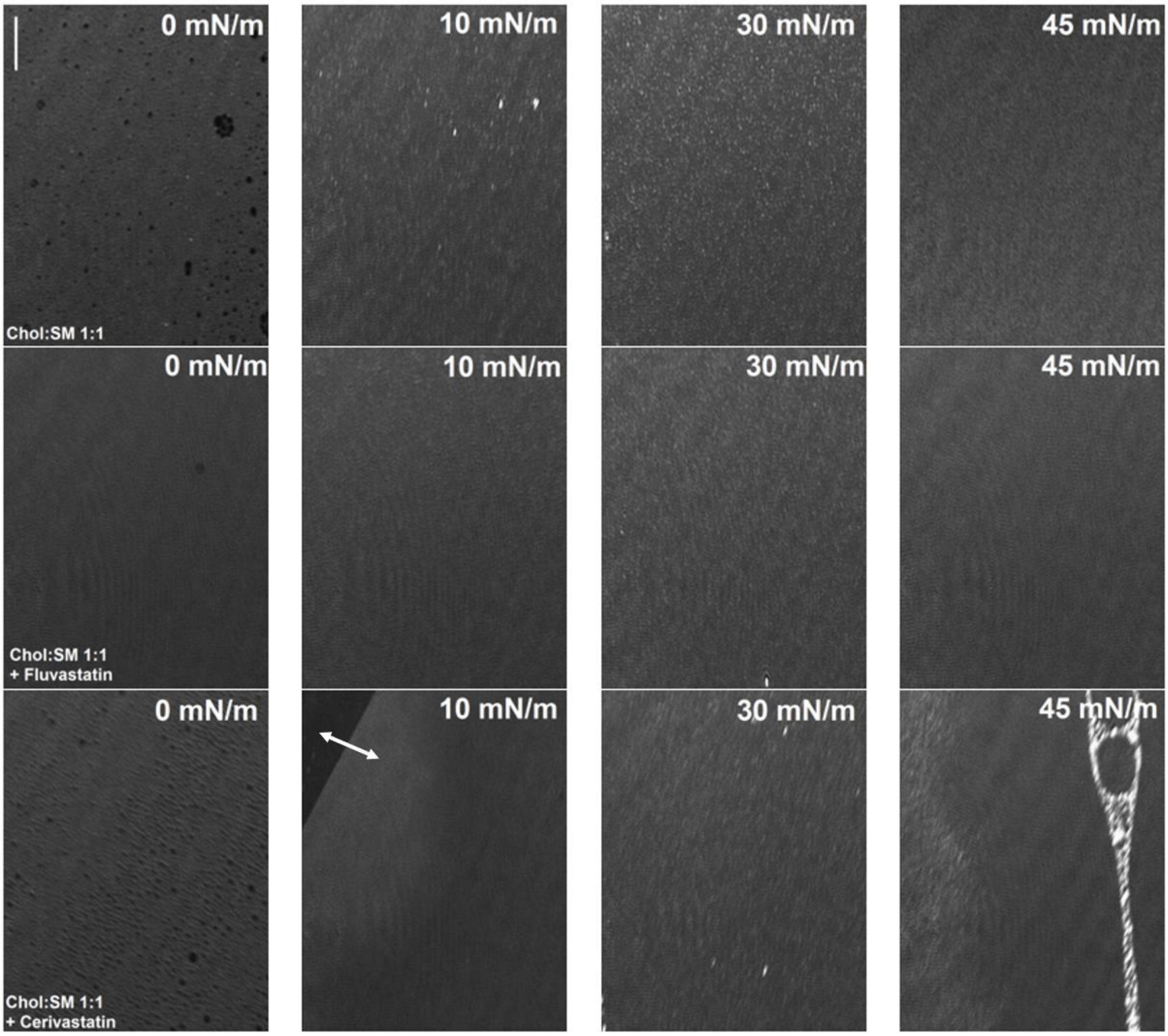

Figure S4. BAM pictures obtained at selected surface pressures for Chol:SM 1:1 monolayers formed on PBS buffer pH 7.4 and PBS pH 7.4 containing 10^{-5} M statins (T=21±1°C). The scale bar is 100 μm.
